# Bartonellosis in a Renal Transplant Recipient: Scratching More Than the Surface

**DOI:** 10.7759/cureus.78514

**Published:** 2025-02-04

**Authors:** José Mário Bastos, Bárbara Pereira, Manuela Bustorff, Ana Rocha, Susana Sampaio

**Affiliations:** 1 Nephrology, Unidade Local de Saúde de Braga, Braga, PRT; 2 Nuclear Medicine, Unidade Local de Saúde de São João, Porto, PRT; 3 Nephrology, Unidade Local de Saúde de São João, Porto, PRT

**Keywords:** bartonellosis, fever of unknown origin, immunosuppression, renal transplantation, zoonotic infections

## Abstract

Fever of unknown origin (FUO) presents a significant diagnostic challenge in renal transplant recipients due to their immunosuppressed state, which predisposes them to a broad spectrum of potential non-infectious and infectious causes, including atypical pathogens. Among these, *Bartonella henselae*, the agent of bartonellosis or cat scratch disease (CSD), is a rare but significant pathogen in this population, capable of causing several systemic manifestations, including hepatosplenic involvement. We describe the case of a 60-year-old male renal transplant recipient who presented with FUO, diarrhea, and hepatosplenomegaly six months post transplantation. The absence of classical features of CSD such as regional lymphadenopathy, along with the initial omission of relevant exposure history, delayed diagnosis. A comprehensive diagnostic workup, guided by a thorough review of history that revealed a cat scratch three weeks prior to presentation, positive polymerase chain reaction (PCR) testing for *B. henselae*, and positron emission tomography-computed tomography (PET-CT) findings of increased splenic uptake with a nodular lesion, corroborated the diagnosis of bartonellosis with splenic involvement. Treatment with azithromycin led to complete resolution of fever and inflammatory markers, and follow-up imaging demonstrated normalization of splenic abnormalities. This case highlights the importance of maintaining a high index of suspicion for zoonotic infections in renal transplant recipients, leveraging advanced diagnostic tools, and tailoring antimicrobial therapy to accommodate immunosuppressive regimens.

## Introduction

Fever of unknown origin (FUO) in solid organ transplant (SOT) recipients represents a significant diagnostic and therapeutic challenge. The immunosuppressive state inherent to these patients increases their susceptibility to opportunistic infections and atypical presentations of common pathogens, which complicates diagnostic efforts and delays effective treatment [1]. The differential diagnosis for FUO in this population includes bacterial, viral, fungal, and parasitic infections, as well as non-infectious causes such as acute rejection, drug fever, and malignancies. Early identification of the underlying cause is critical to improving outcomes, given the heightened risk of sepsis, graft dysfunction, and other complications [2].

Among the infections causing FUO in SOT recipients, *Bartonella henselae *is a rare but important pathogen. It is the causative agent of bartonellosis or cat scratch disease (CSD), a zoonotic infection typically transmitted through scratches, bites, or exposure to saliva from infected cats, including strays or those harboring fleas that serve as vectors for the bacterium [3]. Although less common, transmission has also been associated with flea and tick exposure. In immunocompetent individuals, CSD usually presents with localized lymphadenopathy and a self-limiting clinical course. However, in immunocompromised patients, including transplant recipients, the infection often disseminates, leading to severe systemic manifestations such as prolonged fever, hepatosplenic involvement, endocarditis, osteomyelitis, and central nervous system (CNS) complications [4-6].

The epidemiology of *B. henselae* infection varies geographically, with seroprevalence rates ranging widely depending on climate, cat population density, and exposure to arthropod vectors [4]. While CSD is often self-limiting in immunocompetent individuals, immunocompromised hosts, particularly SOT recipients, are at increased risk of disseminated disease, which can involve multiple organ systems [5].

Risk factors for disseminated *B. henselae* infection in transplant recipients include recent cat exposure, flea infestation, gardening or outdoor activities, and breaches in mucosal barriers, such as those caused by dental procedures [6]. In these patients, the infection can lead to prolonged fever, hepatosplenic involvement, endocarditis, osteomyelitis, and central nervous system (CNS) complications [6].

Hepatosplenic involvement in *B. henselae* infections is characterized by hepatomegaly, splenomegaly, and nodular lesions [6,7]. These abnormalities are frequently detected through imaging modalities such as ultrasound, computed tomography (CT), or positron emission tomography (PET)-CT. Such findings can mimic malignancies, abscesses, or granulomatous diseases, necessitating laboratory confirmation through serological assays or polymerase chain reaction (PCR) testing, which remains the gold standard for definitive diagnosis [7,8].

This report describes the case of a renal transplant recipient with FUO due to *B. henselae* presenting with hepatosplenic involvement. Given the rarity of this infection in transplant recipients, this case reinforces the need for heightened clinical suspicion in the presence of relevant risk factors to ensure timely and effective diagnosis and management. It highlights the diagnostic challenges associated with this condition and underscores the importance of comprehensive history-taking, advanced imaging modalities, and multidisciplinary collaboration. Recognizing this rare entity in transplant recipients is crucial, particularly in the presence of relevant risk factors, to ensure timely and effective diagnosis and management.

## Case presentation

A 60-year-old man presented to the emergency department with a two-week history of fatigue, accompanied by four days of watery diarrhea (approximately six episodes per day) and two days of fever. He denied otalgia, odynophagia, cough, sputum production, chest pain, abdominal pain, vomiting, dysuria, polyuria, oliguria, back pain, or joint pain. He also denied recent travel, high-risk sexual activity, recent dental procedures, gardening or other outdoor activities, contact with pets or stray animals, or initiation of any new medications. His dietary habits adhered to post-transplant recommendations, such as avoiding raw or undercooked foods, unpasteurized dairy, and untreated water sources.

The patient had undergone a deceased brain-dead donor renal transplant with standard criteria six months earlier. Basiliximab was administered for induction of immunosuppression, and his maintenance immunosuppressive regimen included prednisolone (5 mg daily), mycophenolate mofetil (750 mg twice daily), and extended-release tacrolimus (3 mg daily). His medical history was notable for end-stage renal disease secondary to diabetic nephropathy, which required six years of hemodialysis prior to transplantation. He had a 20-year history of type 2 diabetes mellitus with multiple target organ damages, including retinopathy, nephropathy, and peripheral neuropathy. Additionally, he had a 15-year history of hypertension and 12 years of dyslipidemia, and was a former smoker with a 32-pack-year history, having quit 12 years earlier. His non-immunosuppressive medications included NPH insulin, carvedilol, atorvastatin, pantoprazole, and trimethoprim-sulfamethoxazole (960 mg three times weekly).

On physical examination, the patient was alert and well-nourished but showed signs of dehydration. His vital signs revealed an auricular temperature of 38.6°C, blood pressure of 110/60 mmHg, heart rate of 110 beats per minute, and peripheral oxygen saturation of 97% on room air. His oropharynx was clear, with no exudates, and his skin and mucosae were non-icteric and showed no rashes or lesions. No lymphadenopathy was palpable. Cardiovascular examination revealed a regular rhythm without audible murmurs. Pulmonary auscultation revealed symmetrical vesicular breath sounds without adventitious sounds. Abdominal examination was notable for hepatosplenomegaly without tenderness or pain on palpation or percussion.

Initial laboratory investigations (Table 1) revealed elevated inflammatory markers, including leukocytosis with neutrophilia, increased C-reactive protein, and procalcitonin. Acute kidney injury was noted, with a serum creatinine of 2.54 mg/dL, increased from a baseline of 1.32 mg/dL one month earlier, corresponding to an estimated glomerular filtration rate (eGFR) of 28 mL/minute (CKD-EPI). Urinalysis was unremarkable, with no evidence of pyuria, hematuria, or nitrituria. Tests for SARS-CoV-2, influenza, and respiratory syncytial virus were negative. Abdominal ultrasound confirmed splenomegaly (17 cm) and hepatomegaly (19 cm) without identifiable focal lesions or collections. Chest X-ray, renal ultrasound, and Doppler of the allograft showed no abnormalities. The patient was admitted to the renal transplant unit with the diagnoses of FUO, acute kidney injury (presumed to be prerenal due to diarrhea), and hypovolemic hyponatremia. After obtaining three sets of blood cultures and a urine culture, empiric therapy with piperacillin-tazobactam (4.5 g every eight hours, adjusted for renal function) and intravenous fluids (0.9% sodium chloride at 63 mL/hour) was initiated.

**Table 1:** Laboratory results at admission NA: not applicable

Parameter	Units	Patient Value	Reference Range
Hemoglobin	g/dL	13.4	13.0–17.0
Leukocyte count	/µL	14,300	4,000–11,000
Neutrophils	/µL	11,200	1,500–8,000
Lymphocytes	/µL	2,500	1,000–4,000
C-reactive protein (CRP)	mg/L	146	<5
Procalcitonin	ng/mL	1.2	<0.5
Serum creatinine	mg/dL	2.54	0.7–1.3
Blood urea nitrogen (BUN)	mg/dL	105	7–20
Sodium	mmol/L	129	135–145
Potassium	mmol/L	4.1	3.5–5.0
Chloride	mmol/L	100	96–106
Bicarbonate	mmol/L	25	22–28
pH	NA	7.40	7.35–7.45
Lactate	mmol/L	1.1	0.5–2.2
Alkaline phosphatase (ALP)	U/L	87	40–130
Gamma-glutamyl transferase (GGT)	U/L	32	10–71
Lactate dehydrogenase (LDH)	U/L	180	120–250
Aspartate aminotransferase (AST)	U/L	28	10–40
Alanine aminotransferase (ALT)	U/L	35	7–56

By the third day of hospitalization, diarrhea and hyponatremia had resolved, but fever persisted, with two to three spikes daily without a specific pattern, despite fixed paracetamol 1 g every eight hours. Blood and urine cultures collected on admission were negative. Tacrolimus levels measured on the first day of hospitalization were 6 ng/mL (within normal range). Stool studies for virological, bacteriological, parasitological, and *Clostridium difficile* toxin testing were negative. PCR testing for cytomegalovirus (CMV) and Epstein-Barr virus (EBV) was also negative. A computed tomography (CT) scan of the thorax, abdomen, and pelvis confirmed moderate hepatosplenomegaly without other organomegaly, adenopathy, or other abnormalities. A transthoracic echocardiogram revealed no alterations, including vegetation. Given persistent fever, the diagnostic workup was expanded to include repeat blood and urine cultures, supplemented with fungal and mycobacterial testing. A thorough review of the patient’s history prompted him to recall and disclose a scratch by a stray cat three weeks prior. Based on this new information, testing for zoonotic infections, including PCR for *B. henselae, Toxoplasma gondii, Brucella, Leishmania, Coxiella,* and *Leptospira*, was ordered. *B. henselae *PCR returned positive within two days and was confirmed with repeat testing three days later.

**Figure 1:**
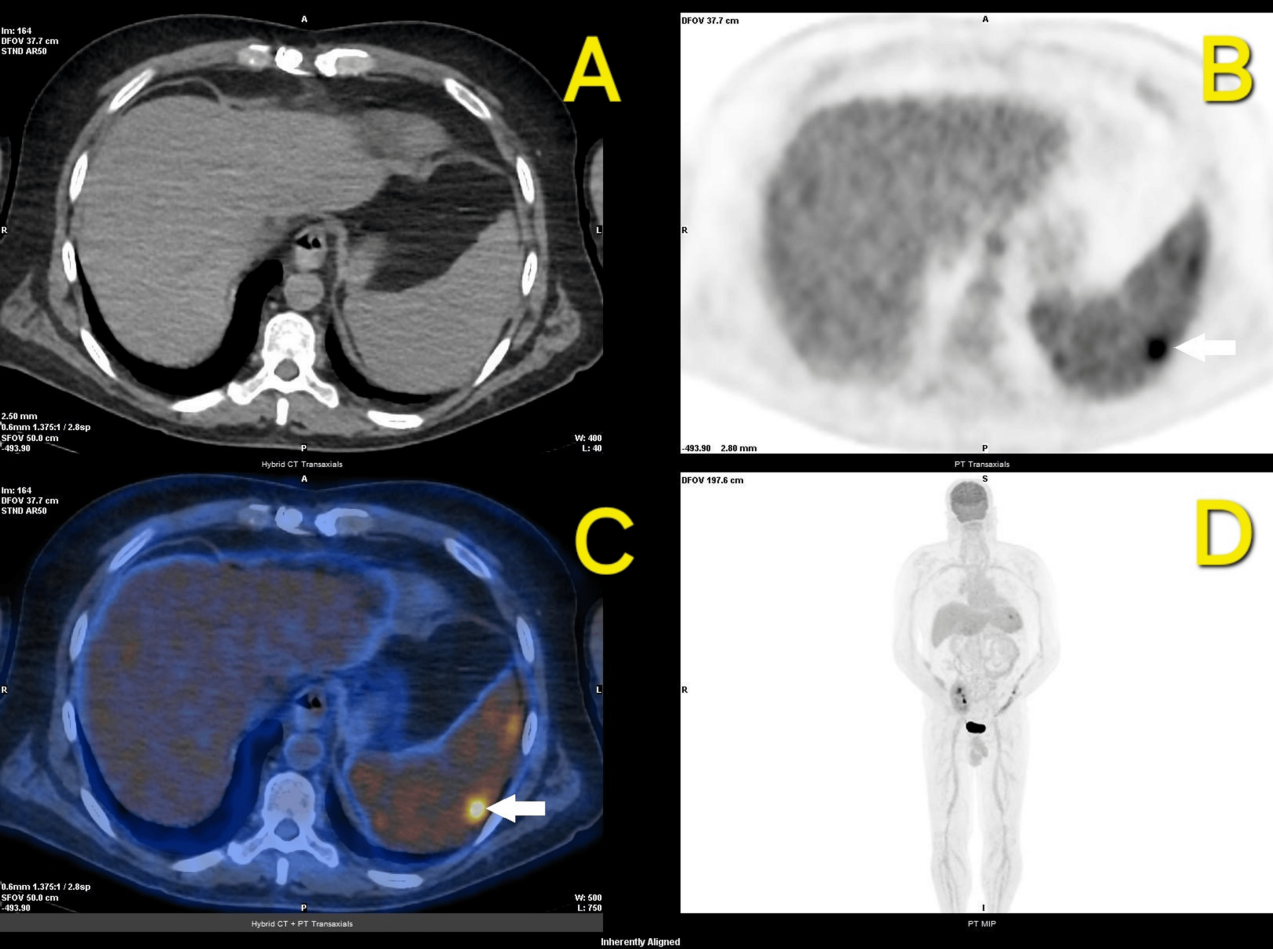
PET-CT with 18F-FDG showing diffusely increased splenic uptake with a nodular, avid lesion in its periphery (white arrows). (A) axial CT; (B) axial PET; (C) axial PET-CT fusion; (D) PET maximum intensity projection (MIP) PET: positron emission tomography;  18F-FDG: fluorodeoxyglucose F 18

With the collaboration of infectious disease specialists, piperacillin-tazobactam was replaced by azithromycin (500 mg daily) on the sixth day of hospitalization. The fever resolved three days after initiating azithromycin, and inflammatory markers improved progressively. A PET-CT performed on the seventh day of hospitalization revealed diffusely increased splenic uptake, highlighting a nodular, avid lesion at its periphery (SUVmax: 7.87) (Figure 1) and reactive thoracic lymph nodes, consistent with an infectious process. The case was discussed with nuclear medicine and infectious diseases physicians, and the splenic nodule was attributed to bartonellosis. Ophthalmological evaluation excluded retinitis, and a transesophageal echocardiogram performed on the 10th day ruled out endocarditis.

The patient was discharged on the 11th day to complete a 14-day course of azithromycin at home. At the three-week follow-up in the infectious diseases clinic, the patient was asymptomatic, with no fever recurrence, normalized inflammatory markers, and resolved hepatosplenomegaly on ultrasound. At the four-week post-discharge follow-up, a repeat PET-CT confirmed the complete resolution of splenic and thoracic abnormalities. On the same day, the patient was evaluated in the nephrology post-transplant clinic and was asymptomatic and afebrile, with renal function at baseline (serum creatinine of 1.3 mg/dL).

## Discussion

CSD, caused by *B. henselae*, typically manifests as a benign and self-limiting illness in immunocompetent individuals. The classical presentation includes localized lymphadenopathy, often accompanied by mild febrile syndrome, fatigue, and malaise following exposure to infected cats through scratches, bites, or contact with flea-contaminated fur [9]. The hallmark feature is tender regional lymphadenopathy near the site of inoculation, which occurs in approximately 90% of cases and is considered a key diagnostic clue [10].

In contrast, immunosuppressed patients, particularly those on chronic immunosuppressive therapy post transplantation, exhibit a markedly different disease phenotype. Blunted inflammatory responses in these individuals often obscure classical signs such as regional lymphadenopathy or inoculation site lesions, as observed in this case. Instead, systemic manifestations dominate, including prolonged fever, hepatosplenic involvement, endocarditis, neuroretinitis, and osteomyelitis [5]. These differences highlight the diagnostic challenges in immunosuppressed patients and emphasize the importance of a high index of suspicion for atypical presentations. In this case, the absence of regional lymphadenopathy and inoculation site lesions, coupled with the initial omission of exposure history with a stray cat, delayed the consideration of CSD.

Persistent fever without a clear focus necessitated a comprehensive diagnostic approach, including an exhaustive clinical history, imaging, and molecular diagnostics. Ultimately, these efforts led to the identification of *B. henselae* DNA through PCR and splenic involvement on PET-CT. Although serologic assays are widely used for the diagnosis of *Bartonella* infections, their sensitivity and specificity can be variable, particularly in immunocompromised patients who may not mount a robust humoral response. In contrast, PCR allows direct detection of *B. henselae* DNA, offering a highly specific diagnostic tool, especially in cases of disseminated infection. However, PCR sensitivity depends on bacterial load and sample type, and its availability is limited in some centers, leading to prolonged turnaround times compared to serology [11]. The discovery of the patient’s history of a cat scratch after persistent inquiry underscores the critical role of detailed history-taking in transplant recipients presenting with FUO. Although PET-CT lacks specificity for differentiating between infectious, malignant, and granulomatous etiologies, its sensitivity in identifying metabolically active lesions makes it an invaluable tool for localizing disease activity in cases of FUO. Studies indicate that PET-CT can detect splenic involvement in up to 30-50% of systemic *Bartonella* infections in immunosuppressed hosts, often revealing findings missed by conventional imaging modalities like CT or ultrasonography [11,12]. In this case, PET-CT was crucial in delineating the extent of splenic involvement and excluding other systemic complications, reinforcing its role in guiding diagnostic and therapeutic strategies.

The decision to initiate azithromycin monotherapy reflected a careful balance between efficacy and safety in the context of the patient’s immunosuppressive regimen. Rifampicin, often used adjunctively in *Bartonella *infections, was avoided due to its significant effects on metabolic enzymes. It induces cytochrome P450 (CYP3A4), reducing plasma levels of tacrolimus, and enhances uridine diphosphate-glucuronosyltransferase (UGT) activity, accelerating the metabolism of mycophenolate mofetil. These interactions could compromise immunosuppressive efficacy and increase the risk of graft rejection [13]. Azithromycin, a macrolide with excellent tissue penetration and minimal interaction with calcineurin inhibitors, demonstrated rapid clinical and biochemical response. The choice of azithromycin is supported by studies highlighting its effectiveness in achieving bacteremia clearance in immunosuppressed patients, particularly in cases with splenic or hepatosplenic involvement [14].

Follow-up PET-CT four weeks post treatment confirmed the resolution of hepatosplenic lesions, reinforcing the diagnosis, and highlighting the importance of imaging in monitoring therapeutic response. Most studies recommend a 14-day antibiotic course for *B. henselae* infections with hepatosplenic involvement, although evidence in transplant populations remains limited [15]. The complete resolution of clinical and radiological abnormalities in this case validates the appropriateness of the chosen regimen, although further studies are needed to establish standardized guidelines for this subgroup of patients.

This case also underscores the indispensable role of multidisciplinary collaboration in managing complex cases. Inputs from nephrology, infectious diseases, and nuclear medicine were pivotal in achieving a timely diagnosis and tailoring treatment to the patient’s unique clinical and pharmacological needs. Future research should focus on optimizing diagnostic algorithms and therapeutic regimens for *B. henselae *infections in transplant recipients, particularly considering the intricate balance required to maintain immunosuppressive efficacy while effectively treating infections.

## Conclusions

This case underscores the importance of maintaining a high index of suspicion for atypical infections in renal transplant recipients presenting with fever of unknown origin. A comprehensive diagnostic approach, including advanced imaging modalities, is essential for achieving a timely diagnosis and initiating appropriate treatment. Multidisciplinary collaboration, particularly involving specialties such as infectious diseases and nuclear medicine, as demonstrated in this case, is equally pivotal in ensuring that therapeutic decisions effectively balance antimicrobial efficacy with the unique challenges posed by immunosuppressive regimens.

The favorable outcome in this case highlights the potential for full recovery, even in complex presentations, when interventions are prompt and precisely targeted. It also underscores the importance of thorough history-taking, as uncovering the critical detail of the patient’s cat scratch exposure ultimately directed both diagnosis and treatment, embodying the essence of “scratching more than the surface” to uncover essential clues.
